# Chemical Properties, Ruminal Fermentation, Gas Production and Digestibility of Silages Composed of Spineless Cactus and Tropical Forage Plants for Sheep Feeding

**DOI:** 10.3390/ani14040552

**Published:** 2024-02-07

**Authors:** Paulo Fernando Andrade Godoi, André Luiz Rodrigues Magalhães, Gherman Garcia Leal de Araújo, Airon Aparecido Silva de Melo, Tiago Santos Silva, Glayciane Costa Gois, Kelly Cristina dos Santos, Daniel Bezerra do Nascimento, Priscila Barreto da Silva, Juliana Silva de Oliveira, Edson Mauro Santos, Thieres George Freire da Silva, Anderson de Moura Zanine, Daniele de Jesus Ferreira, Tadeu Vinhas Voltolini, Fleming Sena Campos

**Affiliations:** 1 Programa de Pós-Graduação em Ciência Animal e Pastagens, Universidade Federal do Agreste de Pernambuco, Garanhuns 55292-270, Pernambuco, Brazil; zootecnia.godoy@yahoo.com.br (P.F.A.G.); andre.magalhaes@ufape.edu.br (A.L.R.M.); airon.melo@ufape.edu.br (A.A.S.d.M.); pribarretosilva23@gmail.com (P.B.d.S.); 2 Setor de Produção Animal, Empresa Brasileira de Pesquisa Agropecuária, Embrapa Semiárido, Petrolina 56302-970, Pernambuco, Brazil; ghermangarcia@hotmail.com (G.G.L.d.A.); tadeu.voltolini@embrapa.br (T.V.V.); 3 Instituto Federal de Educação, Ciência e Tecnologia do Sertão, Ouricuri 56200-000, Pernambuco, Brazil; tiagoifsertao@gmail.com; 4 Programa de Pós-Graduação em Ciência Animal, Universidade Federal do Maranhão, Chapadinha 65500-000, Maranhão, Brazil; anderson.zanine@ufma.br (A.d.M.Z.); flemingcte@yahoo.com.br (F.S.C.); 5 Programa de Pós-Graduação em Zootecnia, Universidade Federal Rural de Pernambuco, Recife 52171-900, Pernambuco, Brazil; kelly_venturosa@hotmail.com (K.C.d.S.); danielbnascimento17@gmail.com (D.B.d.N.); 6 Programa de Pós-Graduação em Zootecnia, Universidade Federal da Paraíba, Areia 58397-000, Paraíba, Brazil; oliveirajs@yahoo.com.br (J.S.d.O.); edsonzootecnista@yahoo.com.br (E.M.S.); 7 Programa de Pós-Graduação em Produção Vegetal, Universidade Federal Rural de Pernambuco, Serra Talhada 56909-535, Pernambuco, Brazil; thieres.silva@ufrpe.br

**Keywords:** crude protein, dry matter, forage conservation, mixed silage, weight gain

## Abstract

**Simple Summary:**

Because the spineless cactus has a low dry matter, neutral detergent fiber and crude protein content, its use alone is not recommended, and it should be combined with other bulky food sources, such as gliricidia, pornunça and buffel grass, as they are found in abundance in semi-arid regions and nutritionally complement the cactus. Thus, a homogeneous mixture of spineless cactus-based silage in combination with these tropical forages can reduce the selection of components by animals, benefiting their performance and reducing costs compared to their conventional diet, given the more efficient intake. This study investigated the chemical composition, fractionation of carbohydrates and proteins, ruminal degradation kinetics and in vitro gas production of silages composed of cactus and tropical forage plants and their potential use as exclusive feed for sheep. Mixed spineless cactus silages with tropical forages have positive effects on ruminal degradation, digestibility and gas production. Mixed spineless cactus silage with tropical forages can be used as exclusive feed for sheep and can replace corn silage in diet composition.

**Abstract:**

The aim was to evaluate the chemical composition, carbohydrates, protein fractionation and in vitro gas production of silages composed of spineless cactus and tropical forages and their effect on sheep performance. Treatments consisted of silages: corn silage (CS), spineless cactus silage (SCS), spineless cactus + gliricidia (SCG), spineless cactus + buffel grass silage (SCBG) and spineless cactus + pornunça (SCP). Silos were opened 60 days after ensiling, and analyses were carried out. The digestibility test lasted for 36 days, with eight animals per treatment. A completely randomized design was adopted. Considering carbohydrate fractionation, CS, SCS and SCBG silages had higher total carbohydrate content (*p* = 0.001). The SCS silage presented a higher A + B1 fraction (*p* = 0.001). The SCBG and SCG silages showed a higher B2 fraction (*p* < 0.0001) compared to the CS and SCS silages. The SCBG and SCP silages presented a higher C fraction (*p* = 0.001). For protein fractionation, the SCP and SCG silages showed higher crude protein contents (*p* = 0.001). The CS and SCS silages showed a higher A fraction (*p* = 0.001). The SCBG silage presented a higher B1 + B2 fraction (*p* = 0.001). The SCG silage showed a higher B3 fraction (*p* = 0.006) compared to SCBG silage. The SCS and SCP silages showed a higher C fraction (*p* = 0.001). Exclusive SCS silage showed higher in vitro dry matter digestibility (*p* = 0.001), dry matter degradability (*p* = 0.001) and total gas production (*p* = 0.001). The use of the SCBG, SCP and SCG silages to feed sheep increased the dry matter intake (*p* < 0.001). Sheep fed the SCG silage showed greater dry matter and crude protein digestibility compared to the sheep fed the CS, SCS and SCP silages (*p* = 0.002). There was a higher water intake (*p* < 0.001) with the use of the SCS and SCG silages to feed the sheep. The SCP and SCG silages provided a greater intake (*p* < 0.001) and excretion (*p* < 0.001) of nitrogen by the animals. Although there were no differences between the treatments for daily gains, lambs that received the spineless cactus-based silage associated with tropical forages showed higher gains (160–190 g/day) than lambs that received CS silage (130 g/day). Thus, the use of spineless cactus associated with buffelgrass, pornunça and gliricidia to prepare mixed silages (60:40) to feed sheep has potential use to feed sheep, with positive effects on nutrient degradation and increases in dry matter intake. Under experimental conditions, we recommend the exclusive use of spineless cactus silage associated with buffel grass, pornunça and gliricidia in feeding sheep in semi-arid regions, as it provides nutrients, water and greater daily gains compared to corn silage.

## 1. Introduction

The increase in the production of spineless cactus (*Opuntia stricta* Haw.) and its use for feeding ruminants demonstrate its importance in arid and semi-arid regions [1]. Its physiology is characterized by a carbon fixation pathway called crassulacean acid metabolism (CAM), in which there is a reduction in water loss due to daytime stomatal closure and nighttime stomatal opening and CO_2_ fixation [2]. In these regions, forage plants are the main source of feed for small ruminants, where there is usually less availability of forage resources during the dry season [3]. Thus, the cultivation and conservation of forages adapted to soil and climate conditions, aiming to meet the feed demand of herds, can increase the efficiency of productive systems [4].

The spineless cactus has some characteristics that are not recommended for ensiling, such as low neutral detergent fiber (259.70 g/kg dry matter), dry matter (g/kg as-fed basis) and crude protein (58.60 g/kg dry matter) contents [5]. Furthermore, the spineless cactus is an excellent source of total digestible nutrients (693.4 g/kg of dry matter Ref. [6]), water-soluble carbohydrates (150.6 g/kg of dry matter [7]) and non-fibrous carbohydrates (555.0 g/kg dry matter [8]). Consequently, spineless cactus has high palatability and passage rate, and it can be consumed in large quantities [9].

The high content of soluble carbohydrates in spineless cactus is fundamental for the fermentative processes during ensiling to develop efficiently, as they are the main substrate for lactic acid bacteria to produce acids, reducing the pH and conserving the ensiled material [8,9,10,11]. However, spineless cactus must be combined with other sources of fiber and crude protein to maintain normal conditions in the rumen and animal performance. In addition, this allows for an adequate synchronization between the energy and nitrogen supply to ruminal microorganisms, considering the high concentration of water-soluble carbohydrates in cactus pear [8]. Ravari et al. [12] reported that making mixed cactus pear and leguminous silages in a semi-arid region improved dry matter and crude protein content and provided a higher dry matter intake and daily gains for goats compared to animals receiving corn silage.

According to Campos et al. [13], some of the forages suitable for this process are the crops adapted to semi-arid regions, such as gliricidia (*Gliricidia sepium* L.), pornunça (*Manihot* spp.) and buffel grass (*Cenchrus ciliaris* L.), as they are found in abundance and for their productive and nutritional potential for feeding ruminants. Thus, the association of spineless cactus with these tropical forage plants in the production of mixed silages can reduce the selection of components by animals, providing better performance and reducing costs compared to conventional diets [9].

Feed and water planning are essential in ruminant production systems in semi-arid regions. For this, it is essential to know the chemical composition, fractions of carbohydrates and proteins and other parameters that optimize the use of tropical forages associated with spineless cactus. However, knowledge of the qualitative characteristics of silage based on spineless cactus is still scarce.

Given the above, the aim was to evaluate the chemical composition, fractionation of carbohydrates and proteins, kinetics of ruminal degradation and in vitro gas production of silages composed of spineless cactus and tropical forage plants and the effect on the productive performance of sheep.

## 2. Materials and Methods

### 2.1. Experimental Site and Ethical Aspects

This experiment was conducted in the Brazilian Agricultural Research Corporation (Embrapa Semiárido), Petrolina—Pernambuco, Brazil. The study was evaluated and approved by the Ethics Committee on the Use of Animals (CEUA) of the Embrapa Semiárido (Opinion no. 0004/2016).

### 2.2. Chemical Analysis and In Vitro Experiment

#### 2.2.1. Experimental Treatments

The experimental design was completely randomized, with five treatments (silages) and four replications per treatment. The treatments consisted of spineless cactus (SC) silage combined with forage plants adapted to the semi-arid region, in a proportion of 60:40, on a dry matter basis: corn silage (CS; Control silage), spineless cactus silage (SCS), spineless cactus + buffel grass silage (SCBG), spineless cactus + pornunça (SCP) and spineless cactus + gliricidia (SCG).

For silage making, buffel grass (*Cenchrus ciliaris* L.) was harvested before the inflorescence period (60–65 days), 0.75 m in height and 15 cm above the ground level. Gliricidia (*Gliricidia sepium*) and pornunça (*Manihot* sp.) were cut 30 cm above the ground, six months after planting, with an average height of 1.70 m, using the tenderest leaves and stems. The spineless cactus Mexican Elephant Ear (*Opuntia stricta* Haw.) was harvested at 24 months after regrowth, and corn (*Zea mays* L.) was harvested 75 days after planting at a height of 15 cm from the ground.

All material was processed through a stationary forage chopper (PP-35, Pinheiro máquinas, Itapira, São Paulo, Brazil), subsequently ensiled, according to treatments, in plastic-drum silos with a capacity of 200 L, and stored for 60 days.

The chemical composition of the silages can be observed in Table 1.

#### 2.2.2. Carbohydrates and Protein Fractionation

Total carbohydrates (TCs) were fractionated according to Sniffen et al. [14], who proposed the fractionation of carbohydrates into four fractions, considering the nutritional availability and the rate of rumen degradation: Fraction A + B1 (considered non-fiber carbohydrates (NFC)); Fraction B2 (composed of the fibrous carbohydrates of the cell wall and the slow ruminal availability, therefore susceptible to the effects of the passage rate); and Fraction C (corresponds to the indigestible NDF - iNDF).

The fraction A + B1 can be expressed by the difference [14]: Fraction A + B1 (%TC) = 100 − (%CP + %EE + %NDFap + %ash) (CP: crude protein, EE: ether extract, NDFap: neutral detergent fiber corrected for ash and protein (constitutes the plant cell wall-soluble carbohydrates, starch and pectin free of ash and protein)).

Fraction B2 was obtained using the following equation [14]: Fraction B2 (%TC) = 100 × ((%NDFap − %NDIP × 0.01 × %CP) − %NDFap × 0.01 × lignin (%NDFap) × 2.4))/% TC, where NDIP is the neutral detergent insoluble protein, and 2.4 is a correction factor described by Sniffen et al. [14].

To determine the iNDF (Fraction C), approximately 5 g of sample was weighed and deposited in non-woven fabric bags (TNT; 100 g/m^2^), which were sealed and incubated in the rumen of two rumen-fistulated sheep. The bags were removed from the rumen after 288 h of incubation, washed intensively in running water, dried in an oven at 105 °C for at least 12 h and weighed. To determine the iNDF, the bags containing residual dry matter were then treated with a neutral detergent solution for 60 min and washed with running water and hot distilled water several times until the residual detergent was completely removed. Subsequently, the bags were immersed in acetone for 5 min, dried in an oven at 105 °C for at least 12 h and weighed [15].

Protein fractionation was calculated using the CNCPS system [14]. The protein was analyzed and calculated for the following fractions: A (non-protein nitrogen—NPN), B1 (soluble protein rapidly degraded in the rumen) + B2 (insoluble protein with intermediate degradation rate in the rumen), B3 (insoluble protein with slow degradation rate in the rumen) and C (insoluble protein, indigestible in the rumen and intestine).

Fraction A was determined by the difference between total nitrogen and nitrogen insoluble in trichloroacetic acid (TCA): Fraction A (%CP) = total nitrogen − nitrogen insoluble in TCA. Fractions B1 + B2 are found in the cellular content and behave in a nutritionally uniform way [16]: Fraction B1 + B2 (%CP) = 100 − (A + B3 + C). The B3 fraction was determined by the following equation: Fraction B3 (%CP) = NDIN − ADIN/total nitrogen × 100.

The levels of insoluble nitrogen in neutral detergent (NDIN) and insoluble nitrogen in acid detergent (ADIN) were determined according to Licitra et al. [17]. Fraction C was considered as acid detergent insoluble nitrogen (ADIN) [14].

Fractions A + B1 (carbohydrate fractionation) and B1 + B2 (protein fractionation) were determined together using the equations. In addition, the laboratory techniques used in this fractionation are simpler, making such procedures more accessible for routine analyses in laboratories.

#### 2.2.3. Degradation Kinetics and In Vitro Digestibility

In vitro degradability was determined according to Tilley and Terry [18], from the in vitro incubation of 600 mg air-dried sample, with 60 mL nutrient medium (composed of buffer solution, pH indicator solution, macro and micro mineral solution, sodium hydroxide solution (1Molar) and reducing solution), prepared according to Goering and Van Soest [19], with pH 6.8 and 15 mL rumen fluid.

The rumen fluid used as inoculum was obtained, jointly and homogenized, from two rumen-fistulated sheep (average body weight of 30.45 kg), kept confined in stalls and fed with elephant grass (*Pennisetum purpureum*) cv. IRI-381 and concentrate based on corn meal and soybean meal, in addition to mineral salt (Ovinofós, Tortura, Porto Alegre, Brazil), with water ad libitum. Ruminal inoculum was filtered through four layers of gauze, injecting carbon dioxide to maintain the anaerobic environment and kept in a water bath (TECNAL, Piracicaba, SP, Brazil) at 39 °C. Samples were incubated at 0, 3, 6, 9, 12, 18, 24, 36 and 48 h, whereas at time zero, the samples underwent only washing under distilled water at 39 °C. The in vitro dry matter digestibility (IVDMD) was determined according to Tilley and Terry [18] and Holden [20],

#### 2.2.4. In Vitro Gas Production

Gas production was determined by in vitro technique with pressure transducer, proposed by Theodorou et al. [21]. Cumulative gas production was estimated by measuring the pressure of the gases produced during the fermentation process, using a pressure transducer (LOGGER AG100–Agricer) and graduated syringes for gas volume, at times 2, 4, 6, 8, 10, 12, 15, 18, 21, 24, 30, 36, 42 and 48 h after incubation.

When the incubation time was reached, fermentation was stopped in an ice bath and subsequently filtered into glass crucibles (with porosity no. 1), previously weighed, with constant washing with distilled water. Differently, the degradation of dry matter was obtained according to the time of incubation. To estimate the parameters a, b and c, the model proposed by Ørskov and Mcdonald [22] was used: PD = a + b (1 – e − ct), where PD = accumulated degradability of the analyzed nutritional component, after a time t; a = range of the degradability curve when t = 0; b = degradability potential of the insoluble fraction of the analyzed nutritional component; c = rate of degradation by fermentative action of fraction b.

The volume of gas was determined by recording the volume of gas displaced into the syringe by moving the syringe plunger until the internal pressure of the bottle returned to ambient pressure, as indicated by a zero reading on the display unit. The time required for the determination of pressures and volumes was relatively short, not exceeding 10–15 s per bottle, and, with that, the temperature remained unchanged during the measurement period. From each reading, the total produced by the bottles without substrate was subtracted for each sample. The equation developed at the Gas Production Laboratory of the Universidade Federal do Agreste de Pernambuco (UFAPE), from 937 observations, in which 1 psi = 4859 mL gas, was used with data observed in psi to obtain the volume of gas produced during the incubations: gas production (mL) = 5.1612 × psi − 0.3017, R^2^ = 0.9873.

Cumulative gas production data were analyzed using the Gompertz two-compartment model [23] using the NLMIXED procedure of Statistical Analysis System [24]: V_t_ = Vf_1_/1 + e(2 − 4kd_1_(t − λ)) + Vf_2_/1 + e(2 − 4kd_2_(t − λ)), where: V_t_ = maximum total volume of gas produced; Vf_1_ = maximum gas volume for the fast-degradation fraction (non-fiber carbohydrates; NFC); Vf_2_ = maximum gas volume for the slow degradation fraction (fibrous carbohydrates; FC); kd_1_ (h) = specific growth rate for the rapid degradation fraction; kd_2_ (h) = specific growth rate for the slow degradation fraction; λ (Lag time) = duration of initial digestion events (latency phase), common to both phases; and t (h) = fermentation time.

### 2.3. In Vivo Experiment

#### 2.3.1. Intake and Digestibility

Forty intact crossbred sheep (7-months-old and 20.0 ± 1.2 kg bodyweight) were distributed in a completely randomized design, with five treatments and eight replications per treatment. The animals were previously identified, weighed, treated against endo- and ectoparasites and housed in individual metabolic cages, provided with feeders and drinking fountain. The cages were previously identified according to the treatment. The experimental period lasted 22 days, with 15 days for adaptation of the animals to silages, cages and feces collection bags and 7 days for data collection.

Silages were offered twice a day, at 09 h and 15 h. Water was provided ad libitum. The leftovers were collected and weighed to determine intake and adjust the dry matter intake (DMI) in order to allow for 10% leftovers in the trough. Feed supplied and leftovers were collected weekly for further laboratory analysis. The DMI and nutrient intake were calculated by the difference between the amount of nutrient present in the feed supplied and the amount of nutrient present in the leftovers, all based on dry matter.

Feces were collected (seven days) using collection bags attached to the animals to determine nutrient digestibility. The bags were weighed and emptied twice daily (08 h 30 and 15 h 30). A subsample of 10% of the total amount of feces from each animal was collected for further analysis. Samples were stored in a freezer at −20 °C until laboratory analyses [25].

#### 2.3.2. Water Balance

Water intake was assessed daily. Water was supplied in buckets, weighed before supply and again 24 h later. The water lost through evaporation was considered when calculating water intake. Water intake via diet was obtained by the difference between the intake of fresh matter and the DM intake of silages. Total water intake (TWI) was evaluated by the following equations: TWI = water intake via drinking fountain (supplied water − evaporated water) + water intake via silage.

#### 2.3.3. Nitrogen Balance

Urine was collected and weighed once a day in plastic buckets containing 100 mL 20% hydrochloric acid (HCl; 2 N) to prevent nitrogen losses through volatilization. A 10% aliquot of the total urine was collected to obtain a composite sample (per animal), packed in identified plastic vials and stored in a freezer at −20 °C. The apparent nitrogen balance (NB) was calculated according to Silva and Leão [26].

#### 2.3.4. Weight Gain

Animals were weighed at the beginning and end of experimental period and after a 12 h period of solid food deprivation (with access to water) to obtain the average daily gain (ADG), bodyweight gain (BWG) and final bodyweight (FBW) according to Nobre et al. [27].

#### 2.3.5. Chemical Analysis

Samples of fresh material, silage after opening the silos, feed supplied, leftovers and feces were pre-dried in a forced ventilation oven at 55 °C for 72 h. The samples were individually processed in a knife mill (Wiley, Marconi, MA 580, Piracicaba, Brazil) with 3 mm mesh sieve to determinate the gas production and in vitro degradability test and with 1 mm mesh sieve to determine the chemical composition. Laboratory analyses were performed using the methods described by AOAC [28] for dry matter (DM), ash, crude protein (CP), ether extract (EE) and acid detergent fiber (ADF). The neutral detergent fiber (NDF) analysis was performed using a heat-stable α-amylase and omitting sodium sulphite (method F-012/1; [29]). The NDF content was expressed exclusive of contaminant ash and protein (NDFap). Total carbohydrates (TCs) were calculated using the equation proposed by Sniffen et al. [14]. The contents of non-fiber carbohydrates (NFCs) were calculated as proposed by Hall [30]. The apparent digestibility coefficient (ADC) of nutrients was calculated as described by Silva and Leão [26].

Total digestible nutrients (TDNs) were estimated on the basis of the data of apparent digestibility and calculated according to Weiss [31]. Metabolisable energy (ME) was estimated according to the NRC [32]: TDN (g/kg) = dCP + dNDFap + dNFC + dEE × 2.25, where dCP = digestible CP; dNDFap = digestible NDFap; dNFC = digestible NFC; and dEE = digestible EE; ME = DE × 0.82, where DE = digestible energy (DE = (TDN/100) × 4.409).

#### 2.3.6. Statistical Analysis

The data were submitted to analysis of variance (ANOVA) using the Statistical Analysis System version 9.1 (SAS University) software, with a significance level of 5%, according to Tukey’s test. The PROC REG procedure of the Statistical Analysis System (SAS, version 9.1) estimated the regression equation between pressure and volume data. In vitro fermentation kinetics and cumulative gas production data were fitted using SAS University PROC NLMIXED and estimated by the least-squares method using the iterative Gauss Newton process. To estimate degradability parameters a, b and c, the model proposed by Ørskov and Mcdonald [22] was used with the aid of the PROC NLIN procedure. Fermentation parameters were generated from data observed at different in vitro incubation times.

The following statistical model was used: Yij = μ + Ti + eij, where Yij = observed value of the dependent variable; μ = overall average; Ti = effect of treatments; and eij = experimental error.

For the digestibility test, the initial body weight of the animals was used as a covariate in the statistical model: Yijk = μ + Ti + β(Xji − X) + eijk, where: Yij = observed value of the dependent variable; μ = overall average; Ti = effect of treatments; β(Xij − X) = effect of the covariate; and eij = experimental error.

## 3. Results

### 3.1. In Vitro Experiment

#### 3.1.1. Carbohydrate Fractionation

CS, SCS and SCBG silages had a higher TC content (*p* = 0.001) compared to SCP and SCG silages. SCS silage presented a higher A + B1 fraction (*p* = 0.001) in relation to the other evaluated silages. SCBG and SCG silages showed a higher B2 fraction (*p* < 0.0001) compared to CS and SCS silages, not differing from SCP silage. SCBG and SCP silages presented a higher C fraction (*p* = 0.001) in relation to the other evaluated silages (Table 2).

#### 3.1.2. Protein Fractionation

SCP and SCG silages showed higher CP content (*p* = 0.001) compared to CS, SCS and SCBG silages. CS and SCS silages showed higher A fraction (*p* = 0.001) compared to SCBG, SCP and SCG silages. The SCBG silage presented a higher B1 + B2 fraction (*p* = 0.001) in relation to the other evaluated silages. SCG silage showed a higher B3 fraction (*p* = 0.006) compared to SCBG silage, not differing from CS, SCS and SCP silages. SCS and SCP silages showed a higher C fraction (*p* = 0.001) compared to CS, SCBG and SCG silages (Table 2).

#### 3.1.3. Degradation Kinetics, In Vitro Dry Matter Digestibility and In Vitro Gas Production

Exclusive SCS showed higher in vitro dry matter digestibility (*p* = 0.001), dry matter degradability (*p* = 0.001) and V_t_ (*p* = 0.001) in relation to the other silages evaluated. Exclusive corn silage showed higher Vf_1_ (*p* = 0.001) in relation to SCBG, SCP and SCG silage but did not differ from the Vf_1_ production of SCS silage. SCP silage presented higher kd_1_ (*p* = 0.001) in relation to the other evaluated silages, not differing from CS silage. SCBG silages showed higher Vf_2_ (*p* < 0.001) in relation to CS, SCS, and SCG silages, not differing from SCP. SCP and SCG silages showed higher kd_2_ (*p* < 0.001) in relation to the other evaluated silages. SCBG silage presented higher λ (*p* = 0.001) in relation to the other evaluated silages (Table 3).

### 3.2. In Vivo Experiment

#### 3.2.1. Intake, Digestibility and Weight Gain

Sheep fed SCBG, SCP and SCG silages had higher DMI (*p* < 0.001), differing from sheep fed SCS silage, which had lower DMI. Sheep that received SCP and SCG silages had higher CP intake (*p* < 0.001) in relation to the sheep that received CS, SCS and SCBG silages. The lowest EE (*p* < 0.001) intake was observed in sheep that received the SCS and SCBG silages. The lowest NDFap (*p* = 0.008) intake was observed in sheep that received the SCS silage. For ADF, sheep that received SCBG, SCP and SCG silages had higher intakes of this component in relation to sheep that received CS and SCS silages (*p* < 0.001) (Table 4).

Sheep fed SCG silage showed greater DM digestibility compared to sheep fed CS, SCS and SCP silages (*p* = 0.002). Sheep fed SCG silage had a higher CP digestibility compared to sheep fed CS, SCP and SCS silages (*p* < 0.001). The lowest NDFap digestibility was obtained for sheep that received CS and SCP silages (*p* = 0.010), differing from sheep that received SCS silage. There was no effect of silages on the final weight (*p* = 0.072), ADG (*p* = 0.255) and BWG (*p* = 0.065) of sheep (Table 4).

#### 3.2.2. Water and Nitrogen Balance

The highest water intake via the drinking fountain was obtained by sheep receiving CS silage (*p* < 0.001). Higher water intake via silage (*p* < 0.001) and total water intake (*p* < 0.001) were obtained by sheep receiving SCG and SCS silages (*p* < 0.001). The highest N intake (*p* < 0.001), N feces (*p* < 0.001) and N balance (*p* < 0.001) were obtained by sheep fed SCP and SGC silages compared to sheep fed CS, SCBG and SCS silages. The highest excretion of N urine (*p* = 0.001) was presented by sheep fed with SCG silage in relation to sheep that received the other evaluated silages (Table 5).

## 4. Discussion

### 4.1. Carbohydrate Fractionation

The types of carbohydrates present in forages directly influence the use of dry matter, since even at high concentrations of total carbohydrates, SCS and SCBG presented higher and lower values for IVDMD and dry matter degradability, which can be explained by the higher content of NFC (611.05 g/kg DM) in SCS, as well as the values of fiber carbohydrates with high contents of NDFap (656.88 g/kg DM) and iNDF (271.99 g/kg DM) of SCBG. In this context, the fractionation of carbohydrates is important in estimating the use of these compounds by ruminal microorganisms [33].

The highest content for Fraction A + B1 corresponds to SCS, because for containing high amounts of soluble sugars added to pectin, it is common for this forage to have a large representation of this fraction. This affects the degradation of carbohydrates, because of the rise in pH that provides cellulolytic microorganisms with more suitable conditions in the rumen [34]. Because of their rapid degradation, these nutrients improve the digestive flow through the gastrointestinal tract, increasing the intake of nutrients [27]. According to Santos et al. [35] and Pessoa et al. [36], feeds with high proportions of fraction A + B1 are great sources of energy for ruminal microorganisms, enabling greater microbial growth.

Fraction B2 occurred in a greater proportion in relation to Fraction A + B1 for all silages, except for SCS. This result is probably due to a high content of fiber carbohydrates, as it is a fraction with a slow rate of ruminal degradation. Fraction B2 showed higher values in SCBG and SCG, which, for the mixed silage, can assist in the balanced supply of energy during the degradation of nitrogen compounds slowly degraded in the rumen.

Fraction C of carbohydrates has an effect on rumen fill, which promotes a lower intake per unit of time, due to its indigestibility [16]. SCBG and SCP presented greater participation of Fraction C (unavailable carbohydrates) of total carbohydrates, confirmed by the high iNDF when compared to the other silages. Management strategies in the production and conservation of forages can minimize the content of unavailable carbohydrates, also reducing their negative effects on animal performance. Thus, the SCS silages obtained lower C fraction values in relation to the other silages, which was possibly due to the high digestibility of fibrous carbohydrates. Thus, the importance of carbohydrate fractions ingested by ruminants is based on the classification of ruminal bacteria regarding the use of carbohydrates that form the plant cell wall and carbohydrates located in the cellular content without structural functions [37].

### 4.2. Protein Fractionation

The high values for the content of soluble protein and NPN contained in Fraction A in SC silage and CS indicate the possibility of using SCS as a source of nitrogen readily available for use by rumen microorganisms, especially when related to high contents of soluble sugars with starch and pectin (Fractions A + B1) of these silages [38].

The lower contents of soluble protein and NPN found in the mixed silages can be justified by the morphological characteristics or by the phenological stage of the species that were ensiled with SCS, which have more fiber constitutions in relation to the other evaluated species. This hypothesis is confirmed by the higher contents of NDF and ADF in the grass and by the NIDIP and ADIP values of the legume and euphorbiacea.

The highest levels of Fractions B1 + B2 resulted in a high percentage of degradable protein in the rumen (DPR). Assessing these levels, SCBG has greater potential in the use of this nitrogen. Several metabolic pathways that allow for the preservation of or alteration in chemical contents are produced during fermentation as a result of the interaction between the chemical properties of the plant and the population of microbes. In forages with high CP content, protein hydrolysis is one of the modifications considered limiting, favoring proteases and an increase in free amino acids and peptides [39]. Thus, the silages under study with the highest percentage of these fractions are considered of superior quality, evidenced by the higher proportion of NPN.

Fraction B3 contains proteins slowly degraded in the rumen; this fraction is composed of insoluble proteins and proteins attached to the cell wall, with a low rate of degradation [40]. Only the silages composed of SCG and SCBG differed from the other silages studied. This divergence is due to the high nitrogen content in the NDF of SCG, as well as the proximity between the nitrogen values contained in the NDF and ADF of SCBG.

In the evaluated silages, the highest contents of iNDF and nitrogen present in the ADF explain the lower use of nitrogen compounds. Much of the energy used by the ruminal microbiota is obtained from the fermentation of dietary carbohydrates. According to Brito et al. [8], the total carbohydrate content in SCS has an average of 808 g/kg DM, which demonstrates proximity to the total carbohydrate concentrations found in the evaluated silages.

### 4.3. Degradation Kinetics, In Vitro Dry Matter Digestibility and In Vitro Gas Production

In relation to in vitro gas production, the fermentation of TC generated larger V_t_ in SCS, which demonstrates the greater availability of energy for ruminal microorganisms. Further, SCG and SCP showed higher values than SCBG for both parameters. This indicates that the highest content of CP influenced IVDMD and DMD, which produced a larger volume of gases after fermentation of the substrates. The lower volume of gases produced by the carbohydrate fermentation of SCBG may have been caused by the fact that 34.41% carbohydrates are present in the fraction that is unavailable.

The ability to ferment and convert carbohydrates into potential energy for ruminants is indicated by the creation of gases. As a result, the ruminal stoichiometry of carbohydrate fermentation (hexoses) results in the creation of short-chain fatty acids; propionate (C3), which is formed in this process but does not produce carbon dioxide, favors a reduction in gas production [41]. Therefore, silages with low contents of non-fibrous carbohydrates and low contents of pectin and starch will provide lower values in the production of propionate, which may interfere with the degradation of carbohydrates, providing a reduced increase in pH, affecting cellulolytic microorganisms in less-adjusted conditions in the rumen.

During the initial events of ruminal degradation, soluble nutrients are responsible for the larger volume of gases produced. The lowest kd_1_ estimated for NFC occurred in SCBG, which allows us to infer that even with a content of Fraction A + B1 of CHO similar to other silages, the high content of NDFap along with the low availability of soluble protein and NPN may have delayed its use by ruminal microorganisms. This is demonstrated by the high latency of 7.82 h, with consequent reductions in total gas volume (V_t_) and maximum gas volume for the fast-digesting fraction (Vf_1_).

The Vf_2_ produced was greater in SCBG, which may have occurred due to the content of Fractions B2 and C, in agreement with the increased levels in the availability of nitrogen compounds, especially proteins with rapid and intermediate degradation in the rumen.

### 4.4. Intake, Digestibility and Weight Gain

The lowest DMI and DM digestibility was registered for sheep fed exclusive cactus silage (0.483 kg/day), not differing from the DMI and DM digestibility presented by sheep that received CS. This fact is due to the low DM content that SCS presents in its composition (175.9 g/kg DM; Table 1) and consequently higher water content. The higher DM intake in SCBG, SCP and SCG silages may be related to the high NDF digestibility in this group. According to [9], diets on a cactus pear basis provide rapid ruminal emptying due to a reduction in NDF and ADF contents, avoiding the limitation of consumption through physical filling. Thus, according to Montgomery and Baumgardt [42], distension of the digestive tract limits feed intake, even if nutritional needs are not met.

The higher intakes of CP and, consequently, of nitrogen, by sheep that received SCP and SCG in relation to the other tested silages are due to the higher CP content of pornunça and gliricidia in relation to the other plants used in the production of silages. Similarly, it was observed that SCP and SCG provided sheep with higher EE intake compared to SCBG and SCS, showing similarity in consumption with sheep that received CS. This result confirms the potential of using these forage species in the composition of silages that will be offered to small ruminants, mainly in combination with spineless cactus [4].

The low concentration of effective fiber and high levels of water and NFC in the SCS stimulated a reduction in DM and NDFap intake, as well as dietary CP intake. Consequently, with less protein available for greater synchronization with soluble carbohydrates, there was a possible reduction in the use of nutrients produced in the rumen, which can be observed by the greater digestibility of DM and CP of silages associated with other tropical forages (Table 4).

The association of a spineless cactus to buffel grass, pornunça and gliricidia in the composition of mixed silages reduces the levels of NDFap and ADF that these plants present in their composition; therefore, the reduction in fibrous fractions contributed to the rapid ruminal emptying, since the spineless cactus does not have enough fiber to limit consumption through physical filling [43], which favored the obtained results. In this sense, we can infer that the supplementation of these forage plants can provide small ruminants with a silage of high nutritional value.

The highest content of NFC in the exclusive cactus pear silage (611.1 g/kg DM; Table 1) in relation to the other silages tested in the sheep’s diet provided a lower DM and CP digestibility for the sheep fed with this silage. Possibly, the higher NFC content and, consequently, lower NDFap content, may have contributed to the reduction in ruminal pH, which reduces the action of the microbial population degrading fibrous carbohydrates and producing microbial protein, resulting in lower digestibility of DM and CP [1]. The values found in SCS were higher than that recommended by Van Soest [16], who recommended, in ruminant feeding, an adequate proportion of non-fibrous carbohydrates for ruminal health and animal performance, with a maximum of 44% NFC in diets for an optimal ruminal function.

Differing from our findings, Pereira et al. [25], when evaluating the digestibility of sheep fed spineless cactus silage with and without inoculant, observed higher DM and CP digestibility coefficients for animals that received spineless cactus silage without an inoculant; however, these authors used urea and ammonium sulfate in their diets, which may have provided greater CP digestibility, given that the nitrogen in these ingredients is readily available. In our study, there was no inclusion of other ingredients in the diets offered to the animals; only roughage was used (tested silages). Thus, we observed that exclusive spineless cactus silages had a high content of indigestible nitrogen compounds (582.5 g/kg CP; Table 1), which may have contributed to a reduction in CP digestibility.

### 4.5. Water and Nitrogen Balance

Animals that received silages containing spineless cactus in their composition had part of their needs in water supplied by this ingredient, reducing the demand for water in the drinking fountains. The results obtained by Magalhães et al. [44] corroborate this information. Working with feedlot sheep, these authors also observed that the water intake decreased with the inclusion of spineless cactus in the sheep diet. The fact that spineless cactus silage and exclusive spineless cactus silage promoted a reduction in water intake via drinking fountains is very positive, especially for animals raised in regions that face prolonged periods of drought, as in the case of a semi-arid region.

Although the SCG and SCS showed similar behavior in relation to water intake via diet and total water intake for the animals, the spineless cactus associated with a legume in the preparation of mixed silages improves the effective fiber and CP contents of the preserved feed, providing a water reserve of high potential. Although spineless cactus is a plant adapted to semi-arid conditions, with potential as a source of water and nutrients for ruminant feed, its use in large proportions or exclusive supply can cause nutritional disorders in ruminant animals [1]. Thus, SCG is a viable alternative for feeding sheep, as it meets the nutritional needs of animals in semi-arid regions.

The N found in feces derives from microbial cells formed in the large intestine, excretion of enzymes and feed that has not been degraded in the gastrointestinal tract. The increase in fecal nitrogen in diets containing SCP and SCG may be related to the attempt to synchronize energy and protein availability for rumen microorganisms. In addition, the SCP and SCG silages presented higher levels of ADIN and ADIP in their composition, which may have contributed to this higher proportion of N excreted in the feces. Despite the higher CP intake obtained by sheep that received SCP and SCG, nitrogen intake by sheep that received SCG was probably higher than necessary to meet the demand, resulting in excess ammonia in the ruminal environment and its subsequent absorption by the ruminal mucosa, which increased the excretion of N in the urine by these animals.

Under experimental conditions, spineless cactus silages have potential for use due to their chemical composition, fractionation of nitrogen compounds and carbohydrates, positive effects on the degradation of nutrients in the rumen, potential digestibility and gas production.

The use of spineless cactus-based silages indicated a greater intake of dry matter and nutrients, lower intake of drinking water and greater metabolic efficiency of proteins in the nitrogen balance. This was similar to corn silage in the performance of sheep in confinement in this experiment. These results confirm the potential for using cactus-based silages, either exclusively or with other tropical forages.

## 5. Conclusions

The spineless cactus associated with buffelgrass, pornunça and gliricidia to prepare mixed silages (60:40) to feed sheep has potential for use in sheep feeding, with positive effects on nutrient intake and digestibility. Under experimental conditions, cactus silage associated with buffelgrass, pornunça and gliricidia can be used as exclusive feed for sheep in semi-arid regions, as it provides sufficient nutrients and water. Furthermore, spineless cactus silage associated with buffelgrass, pornunça and gliricidia maintains the weight gain achieved with corn silage.

## Figures and Tables

**Table 1:** Chemical composition of silages.

Item (g/kg DM)	Silages				
	CS	SCS	SCBG	SCP	SCG
Dry matter *	304.8	175.9	447.2	237.6	215.2
Ash	70.8	95.6	85.2	118.9	89.0
Organic matter	929.2	904.4	914.8	881.1	911.0
Crude protein	85.0	50.0	72.2	130.2	133.8
Ether extract	31.6	19.3	15.0	30.0	30.0
Total carbohydrates	812.6	835.1	827.6	720.9	747.2
Non-fiber carbohydrates	193.3	611.1	170.7	164.3	104.8
NDFap	619.3	224.1	656.9	556.6	642.4
Indigestible neutral detergent fiber	175.2	41.8	272.0	240.4	200.3
Acid detergent fiber	249.1	208.3	315.2	363.3	415.8
NDIP	15.2	14.8	9.9	32.6	31.1
ADIP	11.9	8.8	8.9	27.8	21.6
Total digestible nutrients	634.0	505.8	668.2	610.7	738.3

CS (corn silage); SCBG (spineless cactus + buffel grass silage); SCP (spineless cactus + pornunça); SCG (spineless cactus + gliricidia), SCS (spineless cactus silage); DM (dry matter); NDFap (neutral detergent fiber corrected for ash and protein); NDIP (neutral detergent insoluble protein); ADIP (acid detergent insoluble protein); * in g/kg natural matter.

**Table 2:** Carbohydrate and protein fractionation in silages based on spineless cactus.

Items	Silage					SEM	*p* Value
	CS	SCS	SCBG	SCP	SCG		
	*Carbohydrate fractionation* (g/kg TC)						
TC (g/kg DM)	812.6 ^a^	835.1 ^a^	827.5 ^a^	720.9 ^b^	747.2 ^b^	10.9	0.001
A + B1 (g/kg TC)	237.8 ^b^	731.8 ^a^	206.2 ^b^	228.5 ^b^	140.2 ^c^	49.2	0.001
B2 (g/kg TC)	427.3 ^b^	249.3 ^c^	556.4 ^a^	465.4 ^ab^	583.5 ^a^	59.1	<0.0001
C (g/kg TC)	205.6 ^c^	18.8 ^d^	344.1 ^a^	328.2 ^a^	276.1 ^b^	27.5	0.001
	*Protein fractionation* (g/kg CP)						
CP (g/kg DM)	84.9 ^b^	50.0 ^d^	72.2 ^c^	130.1 ^a^	133.8 ^a^	7.5	0.001
A (g/kg CP)	77.5 ^a^	79.6 ^a^	34.6 ^c^	52.1 ^b^	52.9 ^b^	4.2	0.001
B1 + B2 (g/kg CP)	324.7 ^b^	219.4 ^bc^	507.5 ^a^	111.9 ^c^	171.6 ^c^	33.4	0.001
B3 (g/kg CP)	132.2 ^ab^	118.4 ^ab^	48.2 ^b^	124.7 ^ab^	238.6 ^a^	18.1	0.006
C (g/kg CP)	465.4 ^b^	582.5 ^a^	409.6 ^b^	465.4 ^a^	53.7 ^c^	89.9	0.001

CS (corn silage); SCS (spineless cactus silage); SCBG (spineless cactus + buffel grass silage); SCP (spineless cactus + pornunça); SCG (spineless cactus + gliricidia), *Carbohydrates fractionation*: TC (total carbohydrates); Fraction A + B1 (considered non-fiber carbohydrates (NFC)); Fraction B2 (composed of the fibrous carbohydrates of the cell wall, and of slow ruminal availability, therefore susceptible to the effects of the passage rate); and Fraction C (corresponds to the indigestible NDF-iNDF). *Protein fractionation*: CP (crude protein); A (non-protein nitrogen—NPN), B1 (soluble protein rapidly degraded in the rumen) + B2 (insoluble protein with intermediate degradation rate in the rumen), B3 (insoluble protein with slow degradation rate in the rumen) and C (insoluble protein, indigestible in the rumen and intestine); SEM (standard error of the mean), *p*-value (probability value). ^a,b,c,d^ (average values followed by different letters in the same row are significantly different (*p* < 0.05) by Tukey’s test).

**Table 3:** In vitro dry matter digestibility (IVDMD), dry matter degradability (DMD) and in vitro gas production in corn silage and and silages based on spineless cactus.

Items	Silage					SEM	*p*-Value
	CS	SCS	SCBG	SCP	SCG		
IVDMD	633.9 ^c^	812.2 ^a^	723.2 ^b^	702.3 ^b^	469.7 ^d^	20.7	0.001
DMD	737.2 ^b^	826.6 ^a^	716.4 ^c^	690.0 ^d^	443.6 ^e^	24.4	0.001
V_t_ (mL/g DM)	241.5 ^b^	261.2 ^a^	156.0 ^d^	193.9 ^c^	202.7 ^c^	8.6	0.001
Vf_1_ (mL/g DM)	168.1 ^a^	150.2 ^ab^	32.8 ^d^	131.7 ^bc^	125.3 ^c^	10.9	0.001
kd_1_ (mL/g DM/h)	0.130 ^ab^	0.113 ^b^	0.098 ^c^	0.145 ^a^	0.115 ^b^	0.008	0.001
Vf_2_ (mL)	73.3 ^b^	62.1 ^c^	123.2 ^a^	111.0 ^ab^	77.3 ^b^	11.7	<0.001
kd_2_ (mL/g DM/h)	0.031 ^b^	0.032 ^b^	0.027 ^c^	0.038 ^a^	0.036 ^a^	0.002	<0.001
λ (h)	4.47 ^d^	6.68 ^b^	7.82 ^a^	4.12 ^d^	5.26 ^c^	0.32	0.001

CS (corn silage); SCS (spineless cactus silage); SCBG (spineless cactus + buffel grass silage); SCP (spineless cactus + pornunça); SCG (spineless cactus + gliricidia); V_t_ (maximum total volume of gas produced); Vf_1_ (maximum gas volume for the fast- degradation fraction (non-fiber carbohydrates; NFC)); kd_1_ (specific growth rate for the rapid degradation fraction); Vf_2_ (maximum gas volume for the slow degradation fraction (fibrous carbohydrates; FC)); kd_2_ (specific growth rate for the slow degradation fraction); λ (duration of initial digestion events (latency phase), common to both phases); DM (dry matter); SEM (standard error of the mean); *p*-value (probability value); ^a,b,c,d,e^ (average values followed by different letters in the same row are significantly different (*p* < 0.05) by Tukey’s test).

**Table 4:** Daily intake, coefficient of digestibility of nutrients and weight gain in sheep fed of in silages based on spineless cactus.

Items	Silage					SEM	*p*-Value
	CS	SCS	SCBG	SCP	SCG		
	*Intake* (g/day)						
Dry matter	722 ^b^	483 ^b^	1029 ^a^	1000 ^a^	1078 ^a^	0.11	<0.001
Crude protein	48 ^c^	24 ^c^	82 ^b^	143 ^a^	150 ^a^	0.02	<0.001
Ether extract	29 ^a^	10 ^b^	17 ^b^	33 ^a^	33 ^a^	0.05	<0.001
NDFap	498 ^a^	131 ^b^	679 ^a^	568 ^a^	716 ^a^	0.10	0.008
Acid detergente fiber	158 ^b^	104 ^b^	399 ^a^	359 ^a^	307 ^a^	0.05	<0.001
	*Digestibility* (g/kg)						
Dry matter	610 ^c^	551 ^c^	692 ^ab^	631 ^b^	727 ^a^	30.89	0.002
Crude protein	400 ^dc^	255 ^d^	694 ^ab^	564 ^bc^	789 ^a^	96.70	<0.001
NDFap	685 ^b^	858 ^a^	730 ^ab^	671 ^b^	797 ^ab^	45.50	0.010
	*Weight gain*						
Final bodyweight (kg)	21.4	21.3	21.8	22.1	22.4	0.28	0.072
Bodyweight gain (g)	910	720	1320	1140	1300	0.27	0.065
Average daily gain (g/day)	130	100	190	160	186	0.02	0.255

CS (corn silage); SCS (spineless cactus silage); SCBG (spineless cactus + buffel grass silage); SCP (spineless cactus + pornunça); SCG (spineless cactus + gliricidia); NDFap (neutral detergent fiber corrected for ash and protein); SEM (standard error of the mean), *p*-value (probability value); ^a,b,c,d^ (average values followed by different letters in the same row are significantly different (*p* < 0.05) by Tukey’s test).

**Table 5:** Water intake and nitrogen balance in sheep fed silages based on spineless cactus.

Items (g/day)	Silage					SEM	*p*-Value
	CS	SCS	SCBG	SCP	SCG		
Water intake via drinking fountain	2082 ^a^	353 ^c^	690 ^b^	644 ^b^	534 ^bc^	0.16	<0.001
Water intake via silage	1750 ^c^	4226 ^a^	1012 ^c^	2482 ^b^	3794 ^a^	0.49	<0.001
Total water intake	3832 ^b^	4880 ^a^	1702 ^c^	3126 ^b^	4330 ^a^	0.40	<0.001
N intake	9.81 ^c^	5.06 ^c^	11.89 ^b^	20.84 ^a^	23.07 ^a^	3.40	<0.001
N faeces	4.57 ^b^	2.60 ^c^	4.14 ^b^	7.91 ^a^	5.23 ^a^	0.87	<0.001
N urine	3.35 ^b^	1.52 ^c^	1.95 ^bc^	1.93 ^bc^	5.24 ^a^	0.69	0.001
N balance	18.01 ^c^	17.36 ^c^	48.74 ^b^	49.28 ^a^	56.15 ^a^	8.36	<0.001

CS (corn silage); SCS (spineless cactus silage); SCBG (spineless cactus + buffel grass silage); SCP (spineless cactus + pornunça); SCG (spineless cactus + gliricidia); SEM (standard error of the mean), *p*-value (probability value); ^a,b,c^ (average values followed by different letters in the same row are significantly different (*p* < 0.05) by Tukey’s test).

## Data Availability

The data presented in this study are available on request from the corresponding author.
